# Increasing Compliance of Deep Vein Thrombosis Medical Prophylaxis in Acute Inpatient Rehabilitation Setting

**DOI:** 10.7759/cureus.23134

**Published:** 2022-03-13

**Authors:** Jun Zhang, Olga Komargodski, Andrew McElroy, Claudia Echaide

**Affiliations:** 1 Physical Medicine and Rehabilitation, St. Charles Hospital, Port Jefferson, USA

**Keywords:** compliance, vte prophylaxis, inaptient, rehab, deep vein thrombosis (dvt)

## Abstract

To improve medication reconciliation and decrease the rate of deep vein thrombosis (DVT) after the transfer of brain-injured neurologically impaired patients from an acute hospital setting to an inpatient rehabilitation facility, a performance improvement strategy was put in place. Such a strategy consisted of adding the proper DVT prophylaxis medication and dosage in the preadmission screen to prevent a delay in receiving the appropriate medication. This resulted in a dramatic reduction of inappropriately discontinued medications from 14.2% of patients to 5.78% over six months (p-value: 0.03). However, after the intervention, we surprisingly observed an increased rate of DVT from 6.2% to 10.11% (p-value: 0.03). This increase may be attributable to a larger number of venous duplex studies performed because of increased awareness of venous thromboembolism (VTE).

## Introduction

Venous thromboembolism is a major national health concern and a major contributor to morbidity and mortality as well as significant medical costs [1,2]. More than 90% of thrombosis originates in the deeper veins of the legs [3]. Prophylaxis for deep venous thrombosis (DVT) is an important aspect of the medical management of a patient in an inpatient rehabilitation setting.

Anticoagulation-related adverse events are an important factor in iatrogenic harm [4]. Reducing those events has been The Joint Commission's safety goal since 2008 [5]. Institutions are encouraged to: “Evaluate anticoagulation safety practices, take action to improve practices, and measure the effectiveness of those actions in a time frame determined by the organization”. The Joint Commission also devised a list of consensus of care for anticoagulation communication between hospitals [6].

The occurrence of DVT in stroke patients is 1% [7], however, some studies show incidence as high as 5% [8]. Heparin is suggested as a safe and effective method of DVT prophylaxis in stroke patients [9]. The longer the patient remains off DVT prophylaxis, the higher the risk to develop DVT [10]. In traumatic brain injury patients, the DVT prevalence is even higher at 13% [11], pharmacological prophylaxis is recommended in cases of traumatic brain injury [12]. 

Medication reconciliation is an essential part of the transition of care of all types of patients [13]. Frequently, the documentation is lacking regarding the correct anticoagulation medication and dosing. In a study of 757 patients discharged to the subacute facilities, the discharge summaries contained all the required information on the use of heparin in only 45.4% and warfarin in 16.4% of patients [14]. Previous studies have demonstrated that targeting the reconciliation process reduces medication errors [15].

Lack of information upon transfer from acute hospital care setting to a subacute rehabilitation facility regarding the pharmacological DVT prophylaxis may lead to a delay in receiving the appropriate regimen. By adding the proper medication and dosage in the preadmission screen we can prevent a delay in admitting physician or provider both ordering or continuing and the patient timely receiving their appropriate medication and subsequently prevent DVTs. This research project aims to assess the effectiveness of adding the previous pharmacological deep-vein thrombosis regimen to the preadmission screen to aid the medication reconciliation process.

## Materials and methods

Participants

This is a performance improvement project with Good Samaritan Hospital Medical Center Institutional Review Board (IRB) approval (approval number IRB 19-001) under Category # 5. A chart review of 924 patients was conducted from an inpatient rehabilitation facility. The sample cohort included patients who presented with a cerebral vascular accident, traumatic brain injury, or non-traumatic brain injury. Patients under 18 years old were excluded from the sample. Data was collected at two different points in time. First, from March 2018 to May 2019 a total of 647 patients were included. Secondly, data was collected again from June 2019 to December 2019 and a total of 277 charts were reviewed. The necessary information was gathered from the patient’s electronic medical record.

Patients’ average age was found to be 66 years, they stayed in the inpatient rehabilitation facility for an average of 14 days, and 44% were female while 56% were male. They were discharged home at a rate of 64%, subacute 25%, and acute care 11% (Table 1).

**Table 1:** Demographic of total cohort (N=924) LOS: Length of stay; DC: Discharge

Item	Value
Average age	66.59
Average LOS	14.08
Female	44.33%
Male	55.67%
DC Home	63.96%
DC Acute care	10.71%
DC subacute	25.32%

Design and improvement strategy

The preadmission screen is required documentation of the patient’s condition detailing the patient’s medical and functional status. It is completed within the 48 hours immediately preceding admission to the inpatient rehabilitation. It includes a section with a brief list of the patient’s medications in the acute hospital. However, important information would occasionally be overlooked and medications could sometimes be missed. In May 2019 the preadmission screen was revised and altered to include a separate section to indicate the DVT prophylaxis regimen in the acute hospital including medication type and dosage. To study the effectiveness of this strategy, a total of 924 patient charts were reviewed at two points in time. First, from March 2018 to May 2019 and again from June 2019 to December 2019 after the improvement strategy was placed.

**Figure 1:**
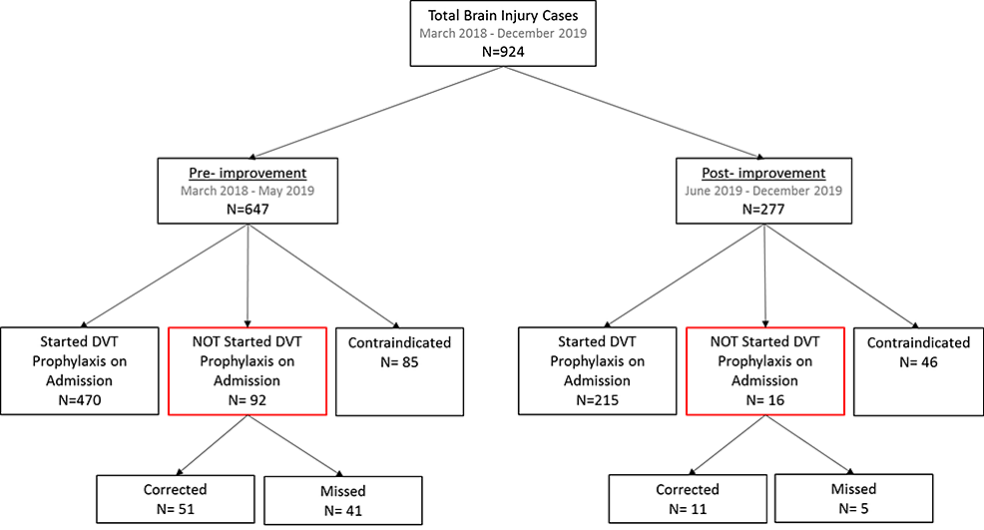
Categorization of sample in groups pre-and post-implementation of the improvement strategy. DVT: Deep vein thrombosis

The goal of the improvement strategy is to increase the rate of pharmacological DVT prophylaxis on admission for patients with the diagnosis of stroke and traumatic brain injury. Those patients frequently have a contraindication for chemical DVT prophylaxis such as recent intracranial hemorrhage. The decision to administer DVT prophylaxis on admission is based on the medication reconciliation list. If this information is not present in the preadmission screen, or if the physician misses the information, the patient will not be started on DVT prophylaxis on admission. Therefore providing the information on the preadmission screen aids in the transfer of care. Based on this analysis three groups were created (Figure 1): (a) patients that were started on pharmacological DVT prophylaxis on admission, (b) patients who did not start on admission, however, the preadmission screen did include DVT prophylaxis among the list of medication. This group was divided further into, (i) those whose medication was corrected, thus they were administered prophylaxis at some point of their stay, (ii) those whose DVT prophylaxis was never started, therefore missed, (c) patients in whom pharmacological DVT prophylaxis was contraindicated and they were never administered DVT prophylaxis on the acute facility they came from.

Statistical analysis

A paired t-test is used to determine a statistically significant difference. 

## Results

A total of 924 patients were admitted to inpatient rehabilitation with a diagnosis of stroke and traumatic brain injury (TBI) between March 2018 and December 2019. Both groups, pre-intervention and post-intervention, did not differ in the following variables: age, gender, length of stay, and discharge disposition (Table 2).

**Table 2:** Pre-and post-improvement control variables PRE: Pre improvement; POST: Post improvement The single p-value for Disposition given as p=0.874

		PRE	POST	p-value
N		647	277	
Average Age		66.03	67.89	0.095
The average length of stay (LOS)		14.15	13.91	0.631
Gender rate	Male	56.88%	49.82%	0.214
	Female	42.81%	45.49%	
Disposition rate	Home	63.99%	63.90%	0.999
	Subacute	25.04%	25.99%	0.823
	Acute care	10.97%	10.11%	0.784

**Figure 2:**
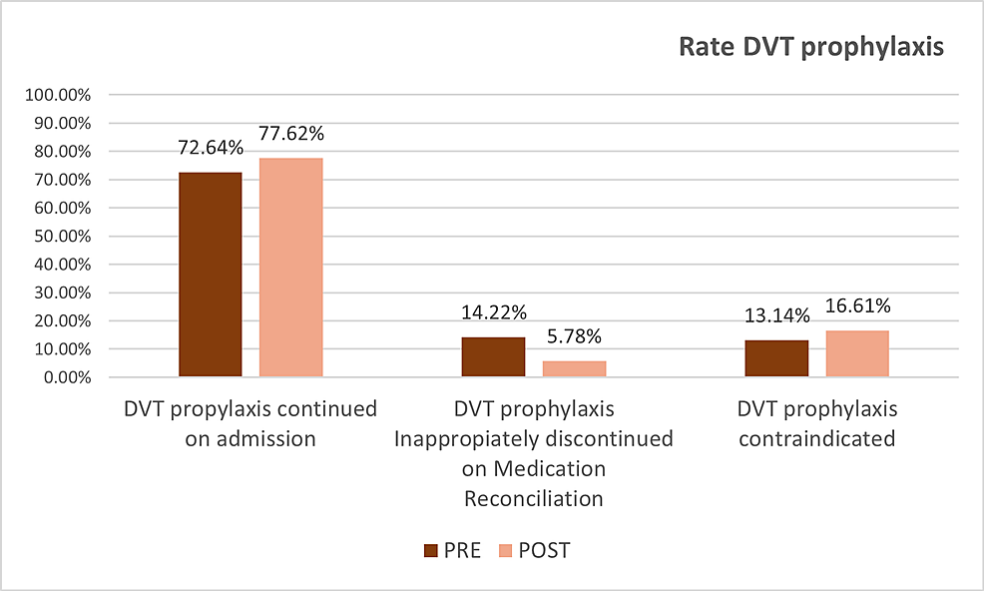
Rate change DVT prophylaxis DVT: Deep vein thrombosis; PRE: Pre improvement; POST: Post improvement

However, after the improvement strategy was put in place there was an increase of patients who continued DVT prophylaxis on admission and a decrease of patients whose DVT prophylaxis was inappropriately discontinued. This result is statistically significant (p=0.003) (Figure 2).

**Figure 3:**
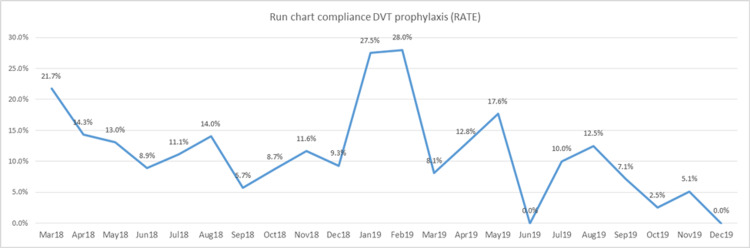
DVT prophylaxis rate of inappropriately discontinued over time DVT: Deep vein thrombosis

Furthermore, this significant decrease is stable over time, since a downward trend is visible when the improvement strategy started in June of 2019 (Figure 3).

When diving deeper into the group who continued DVT prophylaxis from admission versus the group who was inappropriately discontinued, the most used DVT prophylactics were heparin and enoxaparin. Those are also the ones that were discontinued the most on the medication reconciliation (Table 3).

**Table 3:** DVT prophylaxis type DVT: Deep vein thrombosis

Type	Number	Percentage	DVT prophylaxis continued on admission	Percentage	DVT prophylaxis inappropriately discontinued on Medication Reconciliation	Percentage
None	157	17.0%	0	0.0%	26	24.1%
Heparin	404	43.7%	355	51.8%	49	45.4%
Enoxaparin	177	19.2%	154	22.5%	23	21.3%
Coumadin	37	4.0%	33	4.8%	4	3.7%
Noac	86	9.3%	80	11.7%	6	5.6%
Other	63	6.8%	63	9.2%	0	0.0%
Grand Total	924	100.0%	685	100.0%	108	100.0%

Lastly, on the pre-intervention data, there was a DVT rate of 6.20%, which increased to 10.11% post-intervention. This increase is statistically significant (p-value=0.03).

**Table 4:** DVT/PE rate change DVT/PE: Venous thromboembolism

	PRE		POST		P VALUE
DVT/ PE	40	6.20%	28	10.11%	0.03

## Discussion

Our study evaluated whether an improvement of the communication between the acute hospital and rehabilitation facility resulted in better DVT coverage for patients admitted with stroke and traumatic brain injury diagnosis. Restructuring the preadmission screen to include the previous chemical DVT prophylaxis regimen resulted in a dramatic reduction of inappropriately discontinued medications from 14.2% of patients to 5.78% over six months.

The rate of omitted pharmacological DVT prophylaxis from the medication reconciliation was comparable to data shown in other studies. Before the intervention, pharmacological DVT prophylaxis was omitted in 92 of 647 (14.22%) cases. Among all the omitted medications, heparin was the most frequent one, comprising 49 of 92 (45%). Gandara et al. reported rates of heparin omission from the medication reconciliation in 45.4% of total cases [14].

It is to note that after the intervention, an increased rate of DVT was observed from 6.2% to 10.11%. This increase may be attributable to a larger number of venous duplex studies performed as a result of increased awareness of venous thromboembolism (VTE) risk after the quality improvement effort and physician education leading to improved diagnosis of silent DVT, it will need to be further studied to confirm. 

There is a level IIIa recommendation from the Brain Trauma Foundation for TBI patients to use low molecular weight heparin (LMWH) or unfractionated heparin for venous thromboembolism prophylaxis in combination with mechanical prophylaxis. However, there is an increased risk for expansion of intracranial hemorrhage. There is currently insufficient evidence for use of any particular agent [16].

When pharmacological DVT prophylaxis has not been implemented, the prevalence of venous thromboembolism in TBI patients may be as high as 20% [17]. Early chemical DVT prophylaxis appeared not to increase the rate of intracranial hemorrhage [18]. Patients arrive in a rehabilitation facility later in the course of the traumatic injury. Holding a VTE regiment at this period may increase the risk of DVT.

Limitation

The limitation of the current study is the fact that the data was collected in a single facility. Also throughout the study, the major referring acute hospital provided education to staff to include chemical prophylaxis in the discharge summaries. This intervention may have contributed to a reduction in DVT prophylaxis omission and increased the power of the study. Another limitation is the fact that there was no documentation of major bleeding events in the study population. 

## Conclusions

In conclusion, the results of this study suggest that improvement of the preadmission screening including the previous DVT prophylaxis regimen significantly decreased the rate of inappropriately discontinued DVT prophylaxis in TBI and stroke patients. This improvement, however, did not lead to decreased rate of DVT. Additional studies in other settings are needed.
