# Correction: Increasing Compliance of Deep Vein Thrombosis Medical Prophylaxis in Acute Inpatient Rehabilitation Setting

**DOI:** 10.7759/cureus.c60

**Published:** 2022-03-24

**Authors:** Jun Zhang, Olga Komargodski, Andrew McElroy, Claudia Echaide

**Affiliations:** 1 Physical Medicine and Rehabilitation, St. Charles Hospital, Port Jefferson, USA

The submitting author neglected to include their co-authors. Upon publication, Cureus was notified by the submitting author that there were additional authors that should have been included. These authors have now been added:

Olga Komargodski

Andrew McElroy

Claudia Echaide

